# Emotional Intelligence Among Physicians of Different Specialties in Jordan

**DOI:** 10.7759/cureus.90897

**Published:** 2025-08-24

**Authors:** Ahmad M Elheet, Ramzi Alaqtash, Ali M Alhayek, Mohammad H Al-Na'seh, Yahia Albashtawi, Suliman Almohtasib, Mohammad E Al Rafai, Ruba M Jaber

**Affiliations:** 1 Department of Diabetes and Endocrinology, Blackpool Teaching Hospitals NHS Foundation Trust, Blackpool, GBR; 2 Department of Anesthesiology, Jordan University Hospital, Amman, JOR; 3 School of Medicine, The University of Jordan, Amman, JOR; 4 Department of Internal Medicine, Hamad Medical Corporation, Doha, QAT; 5 Department of Orthopedics, Jordan University Hospital, Amman, JOR; 6 Department of Family and Community Medicine, Jordan University Hospital, Amman, JOR

**Keywords:** communication, doctor-patient, ei, emotionality, empathy

## Abstract

Introduction

Emotional intelligence (EI) refers to a set of abilities that enable individuals to recognize and understand emotions, regulate their own responses, maintain motivation, and effectively manage interpersonal interactions. EI plays a crucial role in a physician’s daily practice. Higher EI has been linked to reduced burnout, stronger rapport with patients, improved communication skills, and other professional benefits.

Methodology

Using a cross-sectional observational design, 100 residents and physicians working at the University of Jordan Hospital in 2021 completed a questionnaire that included the Trait Emotional Intelligence Questionnaire-Short Form (TEIQue-SF) to assess global EI and its subscales.

Results

The mean global trait EI was 4.89 ± 0.80. A significant association was found between EI and medical specialty (p = 0.015). Psychiatrists had the highest mean global trait EI, while general surgeons had the lowest. Higher global trait EI was also positively associated with being a specialist. In addition, global trait EI correlated positively with salary, age, and job satisfaction.

Conclusions

The study identified several factors that may influence physicians’ EI levels. Specialty appears to be an important determinant of EI, whereas gender did not predict EI. Further research is needed to explain these findings and to contribute to a broader understanding of EI, both in general and within the context of the doctor-patient relationship.

## Introduction

Emotional intelligence (EI) is defined as the ability to recognize one’s own feelings and those of others, to self-motivate, and to manage emotions both internally and in relationships; it is derived mainly from interpersonal and intrapersonal intelligence, according to Al-Hamdan et al. [1]. Drigas and Papoutsi confirmed that the ability to express one’s own emotions and recognize the emotions of others is an important part of daily human interactions and interpersonal relationships, representing a critical component of human socio-cognitive capacities [2].

EI is essential in every profession but is of particular importance in clinical practice, as it plays a major role in decision-making, patient communication, and the development of strong doctor-patient relationships, thereby allowing for optimal outcomes. Several studies have emphasized the importance of EI in physicians [3-6]. Higher EI in physicians has been associated with a lower incidence of burnout, longer careers, more positive patient-physician interactions, greater empathy, and improved communication skills, as demonstrated by Gorgas et al. and Weng et al. [7,8].

EI encompasses a broad set of skills and attributes that can be learned and developed. Weng et al. found that improving EI in physicians is essential, as it is strongly associated with strengthening the doctor-patient relationship [9]. EI in medical professionals can be improved by raising awareness and offering targeted training sessions [10]. Nelis et al. reported that EI training enhances emotion identification and management skills [10]. Similarly, Satterfield and Hughes determined that emotional skills are teachable and recommended that medical schools integrate EI training into medical education [11]. Boissy et al. further concluded that communication skills training increases patient satisfaction and physician empathy [12].

Effective communication between physicians and patients is essential for delivering high-quality care [13]. Evidence indicates that “a willingness to listen and explain” is considered by patients to be one of the most important attributes of a healthcare professional, underscoring the importance of effective physician-patient communication, as demonstrated in the study by Fellowes et al. [14].

Enhancing communication skills through practice fosters better team environments and improves overall productivity, particularly among healthcare professionals. Physicians should observe and strengthen their EI skills in every interaction with patients, as this promotes effectiveness throughout their careers [11,12]. Among the qualities most valued by patients are strong communication skills, respect, clear explanations, and sufficient time spent during consultations [15-17]. Two studies by Hajibabaee et al. and Raeissi et al. found that physicians with higher EI are better able to apply their social and emotional skills in patient care, similar to findings reported among nurses in healthcare [18,19].

Although good communication skills and high EI are fundamental in all specialties, certain fields, such as psychiatry, pediatrics, general practice, and obstetrics, require greater emphasis on developing EI due to the emotional demands of patient care. Accordingly, part of the training in these specialties should focus on cultivating EI and communication skills. In contrast, specialties with limited direct patient interaction, such as pathology and radiology, may place relatively less emphasis on communication.

The concept of EI in the medical field is an emerging area of research that has gained global interest. Although many studies have examined EI in physicians, it remains insufficiently understood due to numerous confounding factors that are not yet fully explored. Moreover, much of the literature is based on small sample sizes, highlighting the need for larger studies across multiple medical specialties to improve accuracy in defining EI.

We believe there is a lack of knowledge regarding EI among physicians. Few studies have investigated the factors influencing EI, and this study provides an opportunity to explore these factors, raise awareness, and support the integration of EI as a fundamental component of medical and residency training. Additionally, there is a gap in the literature on EI in Jordan, particularly with respect to cross-specialty comparisons.

The aim of this study is to assess EI levels among physicians in different specialties, identify potential factors influencing EI, and propose recommendations to enhance and strengthen EI among physicians. We believe that for physicians to achieve optimal outcomes in medical practice, they must understand EI and undergo training programs to improve their social and emotional skills.

## Materials and methods

Study setting and design

This observational, cross-sectional study was conducted at Jordan University Hospital (JUH) in Amman, Jordan, between January and December 2021. Ethical approval was obtained from the Faculty of Medicine’s Institutional Review Board, and all participants provided informed consent.

Study population and demographics

The target population included all physicians working at JUH during the study period, including residents, specialists, and consultants. Eligible participants were required to be clinically active across any of the available specialties at JUH. Demographic variables collected included age, gender, marital status, level of training (resident vs. specialist), University of Jordan (UJ) graduate status, years of experience, specialty, and monthly salary (in Jordanian dinars).

Inclusion and exclusion criteria

Inclusion criteria were clinically active physicians at JUH practicing in one of the available specialties during the data collection period, including residents, specialists, and consultants. Participants were also required to complete the entire questionnaire and provide consent. Exclusion criteria included incomplete or partially completed surveys, nonclinical faculty, administrative staff, or physicians on prolonged leave during the study period.

Data collection and instruments

Data were collected using an online questionnaire distributed electronically to all eligible participants. The first section of the questionnaire collected sociodemographic variables, including age, gender, marital status, and salary. The second section focused on academic data, including UJ graduate status, training level (resident vs. specialist), specialty, and years of experience.

To assess EI, the study used the Trait Emotional Intelligence Questionnaire-Short Form (TEIQue-SF) developed by Petrides [20]. This self-administered scale is designed to capture personality facets and an individual’s disposition toward emotions. The TEIQue-SF is a 30-item questionnaire derived from the full TEIQue, incorporating all 15 facets by including two items per facet. These facets are organized under four broader factors: well-being, self-control, emotionality, and sociability.

The online form containing the TEIQue-SF was distributed by contacting department heads at JUH, who then forwarded it to the specialists and residents within their departments. Physical paperwork was avoided due to the COVID-19 pandemic.

Data analysis

Participant scores were calculated using the syntax provided with the TEIQue-SF by the psychometrics lab. This syntax generated a global EI trait score and scores for its four components: well-being, self-control, emotionality, and sociability. Internal reliability of the questionnaire was assessed using Cronbach’s alpha.

Scores were grouped by medical specialty to assess differences in trait EI across specialties. Additional analyses examined whether other demographic or professional variables influenced EI scores. One-way ANOVA and bivariate correlation tests were performed using IBM SPSS Statistics for Windows, Version 26.0 (Released 2018; IBM Corp., Armonk, NY, USA). The level of statistical significance was set at α = 0.05.

Ethical considerations

Ethical approval for this study was obtained from the Scientific Research Committee and the Institutional Review Board of the Faculty of Medicine at the UJ (approval number 16/2021). All participants voluntarily consented to participate. To maintain confidentiality, no identifiable personal data, such as names, phone numbers, government identification numbers, or other sensitive information, were collected.

## Results

Sample characteristics

The study included 100 residents and specialists working at JUH, with a mean age of 36.3 years. Of the respondents, 60 (60%) were residents and 40 (40%) were specialists. Male respondents (52%) slightly outnumbered female respondents (48%). More than half of the participants were married (60%). Physicians who graduated from the UJ comprised 60% of the sample, compared with 40% who graduated from other institutions. Among specialties, internal medicine and orthopedics had the highest representation, each accounting for 13% of the sample. Table 1 presents the sociodemographic characteristics of the study participants.

**Table 1:** Sociodemographic characteristics of physicians Monthly salary was recorded in JD. JD, Jordanian dinar; UJ, University of Jordan

Variable		Count (N)	% of Total
Gender	Male	52	52%
	Female	48	48%
Marital status	Single	36	36%
	Married	60	60%
	Engaged	3	3%
	Divorced	1	1%
Level of education	Resident	60	60%
	Specialist	40	40%
Pay status	Paid	84	84%
	Unpaid	12	12%
	Prefer not to say	4	4%
Specialty	Internal medicine	13	13%
	General surgery	12	12%
	Obstetrics and gynecology	12	12%
	Pediatrics	8	8%
	Anesthesia	8	8%
	Family medicine	11	11%
	Radiology	10	10%
	Orthopedics	13	13%
	Psychiatry	2	2%
	ENT	4	4%
	Ophthalmology	4	4%
	Neurosurgery	3	3%
Graduated from the UJ	No	40	40%
	Yes	60	60%
Monthly salary	Unpaid	12	12%
	0-500 JD/month	3	3%
	501-800 JD/month	42	42%
	801-1000 JD/month	3	3%
	1001-1500 JD/month	2	2%
	1501-2000 JD/month	3	3%
	>2000 JD/month	31	31%
	Prefer not to say	4	4%

Reliability and mean scores

For each subscale, several facets determined the mean (± SD) trait EI scores: well-being, 5.20 ± 1.09; self-control, 4.57 ± 1.03; emotionality, 5.00 ± 0.87; and sociability, 4.71 ± 1.01. The mean global trait EI of all participants was 4.89 ± 0.80. Internal consistency of the global trait EI was excellent (Cronbach’s α = 0.90). The alpha coefficients of the four subscales ranged from acceptable (α = 0.63) to good (α = 0.80).

Factors with significant associations and correlations

Table 2 presents the ANOVA results examining the relationship between global trait EI (and its four subscales) with physician specialty and other sociodemographic/academic factors. The analysis showed significant associations between specialty and global trait EI, well-being, and sociability (p = 0.015; p = 0.011; p = 0.028, respectively). Being a specialist was associated with higher mean scores for global trait EI (p = 0.001) and all four subscales (p = 0.002; p = 0.017; p = 0.033; p = 0.025).

**Table 2:** Analysis of associated factors The chi-square statistic is significant at the 0.05 level. EI, emotional intelligence; JD, Jordanian dinar

Variable		Global trait EI (mean ± SD)	p-Value	Well-being (mean ± SD)	p-Value	Self-control (mean ± SD)	p-Value	Emotionality (mean ± SD)	p-Value	Sociability (mean ± SD)	p-Value
Gender			0.18		0.28		0.23		0.98		0.067
	Male	5.00 ± 0.77		5.31 ± 1.09		4.69 ± 0.91		4.99 ± 0.79		4.89 ± 0.98	
	Female	4.78 ± 0.83		5.07 ± 1.08		4.44 ± 1.14		4.98 ± 0.95		4.52 ± 1.01	
Marital status			0.09		0.021		0.169		0.213		0.026
	Divorced	5.27 ± 0		6.00 ± 0		4.83 ± 0		5.63 ± 0		4.00 ± 0	
	Married	5.09 ± 0.78		5.45 ± 1.06		4.73 ± 1.05		5.11 ± 0.87		4.95 ± 0.93	
	Engaged	5.03 ± 0.44		5.11 ± 0.25		4.83 ± 1.01		5.13 ± 0.57		4.72 ± 0.69	
	Single	4.54 ± 0.75		4.76 ± 1.07		4.26 ± 0.98		4.75 ± 0.86		4.33 ± 1.06	
Level of education			0.001		0.002		0.017		0.033		0.025
	Specialist	5.22 ± 0.72		5.60 ± 0.98		4.87 ± 0.85		5.21 ± 0.82		4.99 ± 0.92	
	Resident	4.67 ± 0.78		4.93 ± 1.08		4.37 ± 1.10		4.84 ± 0.88		4.53 ± 1.03	
UJ Graduate			0.031		0.102		0.471		0.035		0.018
	Yes	5.03 ± 0.82		5.34 ± 1.00		4.63 ± 1.12		5.14 ± 0.90		4.91 ± 1.04	
	No	4.68 ± 0.73		4.98 ± 1.19		4.47 ± 0.88		4.76 ± 0.77		4.42 ± 0.89	
Specialty			0.015		0.011		0.56		0.15		0.028
	Psychiatry	5.62 ± 0.12		5.92 ± 0.82		5.08 ± 0.59		5.44 ± 0.44		5.83 ± 0.47	
	ENT	5.57 ± 0.32		5.71 ± 0.53		5.17 ± 0.24		5.75 ± 0.64		5.63 ± 0.42	
	Anesthesia	5.43 ± 0.38		6.06 ± 0.46		5.06 ± 0.73		5.23 ± 0.57		5.17 ± 0.91	
	Internal medicine	5.39 ± 0.86		5.83 ± 1.08		4.82 ± 1.36		5.42 ± 0.90		5.37 ± 1.15	
	Ophthalmology	5.34 ± 0.79		5.83 ± 1.16		5.08 ± 0.52		5.41 ± 1.10		4.67 ± 1.13	
	Family medicine	4.81 ± 0.96		5.53 ± 0.95		4.42 ± 1.15		4.93 ± 1.18		4.24 ± 0.96	
	Obstetrics and gynecology	4.78 ± 0.67		4.82 ± 0.95		4.32 ± 1.28		5.04 ± 0.90		4.69 ± 1.14	
	Orthopedics	4.69 ± 0.60		4.82 ± 0.98		4.64 ± 0.57		4.59 ± 0.73		4.59 ± 0.63	
	Pediatrics	4.62 ± 0.92		4.63 ± 0.90		4.56 ± 1.15		4.78 ± 0.82		4.58 ± 1.17	
	Radiology	4.53 ± 0.69		4.67 ± 1.02		4.22 ± 0.82		4.99 ± 0.74		4.08 ± 0.68	
	Neurosurgery	4.52 ± 0.63		4.83 ± 1.32		3.94 ± 0.67		4.58 ± 0.51		4.67 ± 0.87	
	General surgery	4.49 ± 0.84		4.82 ± 1.34		4.25 ± 1.21		4.54 ± 0.77		4.43 ± 0.93	
Salary			0.009		0.095		0.306		0.09		0.035
	Unpaid	4.47 ± 0.63		4.71 ± 1.02		4.44 ± 0.65		4.58 ± 0.89		4.17 ± 0.69	
	0-500 JD/month	5.60 ± 0.09		5.67 ± 0.73		5.06 ± 0.42		5.75 ± 0.63		5.61 ± 0.51	
	501-800 JD/month	4.65 ± 0.83		4.93 ± 1.23		4.32 ± 1.24		4.83 ± 0.88		4.49 ± 1.05	
	801-1000 JD/month	5.10 ± 0.32		5.50 ± 0.44		4.22 ± 1.11		5.38 ± 0.33		5.33 ± 1.30	
	1001-1500 JD/month	4.78 ± 0.97		5.17 ± 1.41		5.08 ± 0.59		4.44 ± 0.80		4.17 ± 1.18	
	1501-2000 JD/month	5.12 ± 0.81		5.50 ± 1.64		4.44 ± 0.42		5.00 ± 0.88		5.22 ± 1.06	
	>2000 JD/month	5.24 ± 0.67		5.61 ± 0.92		4.88 ± 0.80		5.23 ± 0.73		5.03 ± 0.95	

Graduation from the UJ was associated with higher mean scores in global trait EI, emotionality, and sociability (p = 0.031; p = 0.035; p = 0.018, respectively). Marital status also showed significant associations with well-being and sociability (p = 0.021; p = 0.026, respectively), with married and engaged physicians scoring higher. No significant associations were found between gender and any of the EI subscales.

Table 3 presents the results of the bivariate correlation analysis examining associations between trait EI and selected factors. A positive correlation was found between age and global trait EI (r = 0.342, p < 0.001). Age also showed positive correlations with three subscales: well-being (r = 0.313, p = 0.002), self-control (r = 0.237, p = 0.013), and sociability (r = 0.286, p = 0.004). Additionally, global trait EI and all four subscales, well-being, self-control, emotionality, and sociability, were positively correlated with job satisfaction (r = 0.469, p < 0.001; r = 0.455, p < 0.001; r = 0.283, p = 0.004; r = 0.392, p < 0.001; r = 0.309, p = 0.002, respectively).

**Table 3:** Bivariate correlations between scale variables The chi-square statistic is significant at the 0.05 level. EI, emotional intelligence

Variable		Global trait EI	Well-being	Self-control	Emotionality	Sociability
Age	r	0.342	0.313	0.237	0.188	0.286
	p	<0.001	0.002	0.013	0.062	0.004
Job satisfaction	r	0.469	0.455	0.283	0.392	0.309
	p	<0.001	<0.001	0.004	<0.001	0.002
Monthly salary	r	0.336	0.297	0.197	0.220	0.262
	p	0.001	0.003	0.054	0.031	0.010

When analyzing the relationship between physicians’ salaries and their EI scores, four respondents who chose not to disclose their salaries were excluded. ANOVA tests revealed a significant association between higher monthly salaries and global trait EI, as well as the sociability subscale (p = 0.009; p = 0.035, respectively). Bivariate correlation analysis further showed a positive correlation between monthly salary and global trait EI (r = 0.336, p = 0.001). In addition, well-being, emotionality, and sociability scores were positively correlated with physicians’ salaries (r = 0.297, p = 0.003; r = 0.220, p = 0.031; r = 0.262, p = 0.010, respectively).

## Discussion

The aims of our study were to measure the mean global trait EI of residents and specialists across different specialties in the JUH, to examine differences between participants, to explore various sociodemographic and professional factors that may influence a physician’s EI, and to propose recommendations to enhance EI among physicians. The mean global trait EI for the entire sample was 4.89 ± 0.80.

The analysis revealed a significant association between a physician’s specialty and global trait EI (p = 0.015). The three specialties with the highest mean global trait EI were psychiatry (5.62 ± 0.12), ENT (5.57 ± 0.32), and anesthesia (5.43 ± 0.38). The higher EI score among psychiatrists is expected, as a cornerstone of psychiatric practice is exploring patients’ backgrounds and encouraging them to confront their emotions while simultaneously managing their own. Furthermore, Louie et al. concluded that self-knowledge is a fundamental principle of psychotherapy, as it is essential for understanding one’s patients [21].

In contrast, the three specialties with the lowest mean global trait EI were general surgery (4.49 ± 0.84), neurosurgery (4.52 ± 0.63), and radiology (4.53 ± 0.69). Compared with other physicians, surgeons are exposed daily to several factors that may negatively affect EI, including lack of sleep, long working hours, poor job satisfaction, and strained interpersonal relationships [3]. It is important to note that higher EI levels can enhance performance in the operating theater through improved situational awareness, communication, decision-making, leadership, and teamwork [3]. Radiologists, on the other hand, often focus more on technical medical issues than on patients’ values and perspectives. Similarly, Cannavale et al. explained the low EI scores observed in this specialty [22].

The study findings revealed that age, monthly salary, and job satisfaction were positively correlated with global trait EI (r = 0.342, p < 0.001; r = 0.336, p = 0.001; r = 0.469, p < 0.001, respectively). Physicians’ experience in handling patients’ emotions, improving communication skills, and strengthening doctor-patient relationships tends to increase with age. The data support this, showing that sociability was positively correlated with age (r = 0.286, p = .004); this finding is also consistent with Weng et al.’s 2008 study [9]. Younger physicians, particularly residents, are often less experienced in effective communication with patients, usually acquiring these skills over time from more senior colleagues. Both Weng et al. [9] and Mayer et al. [23] have shown that EI skills develop with experience.

A higher salary was correlated with higher global trait EI scores (r = 0.336, p = 0.001), as well as with higher scores in well-being, emotionality, and sociability (r = 0.297, p = 0.003; r = 0.220, p = 0.031; r = 0.262, p = 0.010, respectively). Similar findings were reported by Sjöberg in 2008, who observed an association between higher EI and higher income levels [24]. Greater job satisfaction also contributes to higher EI, as physicians who are more content with their professional lives typically experience less burnout and stress while demonstrating improved social and emotional abilities [3]. Our data confirmed this, showing that job satisfaction positively correlated with sociability (r = 0.309, p = 0.002) and emotionality (r = 0.392, p < 0.001).

The presence of only one divorced participant in our sample skewed the results, suggesting an association between divorce and higher global trait EI. This was due to the divorced participant’s score being higher than the mean scores of single, engaged, and married participants. Nevertheless, there was a significant association between marital status and the subscales of well-being (p = 0.021) and sociability (p = 0.026). Married and engaged participants had significantly higher mean scores compared with single participants. For married participants, the mean well-being and sociability scores were 5.45 ± 1.06 and 4.95 ± 0.93, respectively, while for single participants, they were 4.76 ± 1.07 and 4.33 ± 1.06, respectively. These higher scores may be explained by the emotional and social support provided by a partner, which enhances happiness, mental health, and social well-being, as suggested by Braithwaite and Holt-Lunstad’s study [25]. Marriage also entails responsibility toward one’s family, and the feelings of love and care associated with this responsibility may further explain the observed results.

Interestingly, UJ graduates were associated with higher mean scores in global trait EI (p = 0.031). One possible explanation is that UJ medical students take a course in family medicine, which orients them toward patient-centered care. As Johnson’s study demonstrated, EI teaching facilitates specific aspects of the doctor-patient relationship and should therefore be considered a priority in medical education [26]. Another possible explanation is familiarity with the workplace, as UJ graduates complete at least three years of clinical training at JUH, which may enhance their adaptability compared with graduates from other universities. Nonetheless, further research is needed to investigate and clarify these associations.

In this study, the mean global trait EI score among doctors was 4.89 ± 0.80, which is very close to that reported by Al Huseini et al. in Oman (4.20 ± 0.42) in a study of 320 residents from various specialties [27]. Consistent with the findings of Lin et al. [5] and McKinley et al. [28], this study found no significant difference in mean global trait EI scores between genders. In Lin et al.’s study [5], which included only general surgery residents, the mean global trait EI score was 5.18 ± 0.81, compared with 4.49 ± 0.84 for general surgeons in our study. In line with our results, Weng et al. [9] also reported a positive association between age and global trait EI. By contrast, Bharamanaikar and Kadadi [29] found no significant associations between EI scores and either age or length of service.

In our study, the positive correlation between age and global trait EI, together with the significant difference observed between residents and specialists in their mean global trait EI scores (p = 0.001), indicates that work experience plays a role in shaping EI, supporting the findings of Weng et al. (2008) [9].

Although this sample size represented only 18% of the total physician population, it was sufficient to detect a statistically significant difference in EI across medical specialties, as demonstrated by the ANOVA test (p = 0.015). While an ideal a priori power analysis might have suggested a larger sample to detect smaller effect sizes with 80% power, the observed statistically significant results indicate that the current sample was adequate to identify meaningful differences. These findings, therefore, provide a solid foundation for future research with larger and more balanced samples.

We recommend the implementation of interventions at various stages of physicians’ careers to improve EI. Such interventions should target medical students, residents, and specialists. The first step is to raise awareness among physicians about the importance of EI through awareness campaigns. This should be followed by structured EI training sessions to enhance physicians’ abilities in emotional identification and management, as shown to be effective by Nelis et al. [10] and Boissy et al. [12]. In addition, establishing a physician rating system, specifically one that evaluates communication skills, could help physicians identify areas of weakness. Linking these ratings to incentives may further encourage improvement. Periodic assessments of EI and communication skills should also be implemented to ensure sustained progress and a consistently high standard of care. Finally, systemic measures such as increasing income and reducing workload would contribute not only to improving EI but also to enhancing physicians’ overall well-being.

This study has several limitations. The sample size was relatively small, with only 100 physicians participating (17% of JUH physicians). Most participants were residents from different specialties who had similar salaries but varying EI scores; other potential confounders, such as work hours, may have contributed to the differences observed between specialties. Moreover, the COVID-19 pandemic created challenges in accessing physicians, and the psychological stress experienced during this period may have influenced the results.

## Conclusions

The mean global trait EI in our sample was 4.89 ± 0.80. Factors significantly associated with EI among physicians included age, level of education, monthly salary, and marital status, while gender showed no association. EI also varied between specialties, with psychiatry, ENT, and anesthesia demonstrating the highest mean scores.

We recommend that future research examine EI differences among physicians within the same specialty but with varying salaries in order to better evaluate the influence of work nature and conditions. Increasing the sample size in future studies will help achieve more robust and accurate results. Our findings also support the integration of EI-focused training and education into medical practice. Finally, incorporating patients’ perspectives on physician EI may provide valuable insights and contribute to a deeper understanding of its role in clinical care.

## Appendices

Questionnaire

Please find attached the questionnaire used in this study.

We are a group of researchers aiming to measure the emotional intelligence of residents and specialists of different specialties and see if there are differences between specialties regarding how emotionally intelligent the physicians are. No personal information will be required. By continuing with this questionnaire, you consent to participate in our study. This questionnaire will only take five to seven minutes of your time.

Gender:                      Male                                   Female

Age:  _______ years old

Did you graduate from the University of Jordan?

                          Yes                               No

If you are a specialist, how many years have gone by since becoming a specialist?

___________ years

What specialty are you in?

Please answer the following questions in the table below:

**Table 4:** Emotional Intelligence Questionnaire Part 1

Variable	Categories
Marital status	Single, Married, Engaged, Divorced, Widowed, Prefer not to say
Year of residency	First, Second, Third, Fourth, Fifth, Sixth, Seventh
Monthly salary	Unpaid, 1-500 JD, 501-800 JD, 801-1000 JD, 1001-1500 JD, 1501-2000 JD, More than 2000 JD, Prefer not to say
Job satisfaction	1 (very dissatisfied) to 5 (very satisfied)

**Table 5:** Emotional Intelligence Questionnaire Part 2 Instructions: Please answer each statement below by putting a circle around the number that best reflects your degree of agreement or disagreement with that statement. Do not think too long about the exact meaning of the statements. Work quickly and try to answer as accurately as possible. There are no right or wrong answers. There are seven possible responses to each statement ranging from 'Completely Disagree' (number 1) to 'Completely Agree' (number 7). Trait Emotional Intelligence Questionnaire - Short Form (TEIQue-SF). This 30-item form includes two items from each of the 15 facets of the TEIQue. Items were selected primarily on the basis of their correlations with the corresponding total facet scores, which ensured broad coverage of the sampling domain of the construct. The -SF can be used in research designs with limited experimental time or wherein trait El is a peripheral variable. Although it is possible to derive from it scores on the four trait El factors, in addition to the global score, these tend to have somewhat lower internal consistencies than in the full form of the inventory. The -SF does not yield scores on the 15 trait EI facets. Scoring information for the TEIQue-SF is available at http://www.psychometriclab.com/Home/Default/14. Please note that we cannot provide any advice on how to run the syntax in SPSS or other statistical software. Please make sure you read the FAQ section at http://www.psychometriclab.com/Home/Default/18. In particular, note that we do not provide free information regarding norms or free feedback reports. Norms and reports are available for a fee (email admin@teique.com for quotes). Source: Petrides (2009) [30]

1. Expressing my emotions with words is not a problem for me.	1	2	3	4	5	6	7
2. I often find it difficult to see things from another person's viewpoint.	1	2	3	4	5	6	7
3. On the whole, I'm a highly motivated person.	1	2	3	4	5	6	7
4. I usually find it difficult to regulate my emotions.	1	2	3	4	5	6	7
5. I generally don't find life enjoyable.	1	2	3	4	5	6	7
6. I can deal effectively with people.	1	2	3	4	5	6	7
7. I tend to change my mind frequently.	1	2	3	4	5	6	7
8. Many times, I can't figure out what emotion I'm feeling.	1	2	3	4	5	6	7
9. I feel that I have a number of good qualities.	1	2	3	4	5	6	7
10. I often find it difficult to stand up for my rights.	1	2	3	4	5	6	7
11. I'm usually able to influence the way other people feel.	1	2	3	4	5	6	7
12. On the whole, I have a gloomy perspective on most things.	1	2	3	4	5	6	7
13. Those close to me often complain that I don't treat them right.	1	2	3	4	5	6	7
14. I often find it difficult to adjust my life according to the circumstances.	1	2	3	4	5	6	7
15. On the whole, I'm able to deal with stress.	1	2	3	4	5	6	7
16. I often find it difficult to show my affection to those close to me.	1	2	3	4	5	6	7
17. I'm normally able to "get into someone's shoes" and experience their emotions.	1	2	3	4	5	6	7
18. I normally find it difficult to keep myself motivated.	1	2	3	4	5	6	7
19. I'm usually able to find ways to control my emotions when I want to.	1	2	3	4	5	6	7
20. On the whole, I'm pleased with my life.	1	2	3	4	5	6	7
21. I would describe myself as a good negotiator.	1	2	3	4	5	6	7
22. I tend to get involved in things I later wish I could get out of.	1	2	3	4	5	6	7
23. I often pause and think about my feelings.	1	2	3	4	5	6	7
24. I believe I'm full of personal strengths.	1	2	3	4	5	6	7
25. I tend to "back down" even if I know I'm right.	1	2	3	4	5	6	7
26. I don't seem to have any power at all over other people's feelings.	1	2	3	4	5	6	7
27. I generally believe that things will work out fine in my life.	1	2	3	4	5	6	7
28. I find it difficult to bond well even with those close to me.	1	2	3	4	5	6	7
29. Generally, I'm able to adapt to new environments.	1	2	3	4	5	6	7
30. Others admire me for being relaxed.	1	2	3	4	5	6	7
